# Unique inducible filamentous motility identified in pathogenic *Bacillus cereus* group species

**DOI:** 10.1038/s41396-020-0728-x

**Published:** 2020-08-07

**Authors:** Martha M. Liu, Shannon Coleman, Lauren Wilkinson, Maren L. Smith, Thomas Hoang, Naomi Niyah, Manjari Mukherjee, Steven Huynh, Craig T. Parker, Jasna Kovac, Robert E. W. Hancock, Erin C. Gaynor

**Affiliations:** 1 Department of Microbiology and Immunology, The University of British Columbia, Vancouver, BC Canada; 2 Department of Food Science, The Pennsylvania State University, University Park, PA USA; 3 Produce Safety and Microbiology Unit, Western Region Research Center, USDA, Agricultural Research Service, Albany, CA USA

**Keywords:** Bacterial physiology, Transcriptomics, Bacteriology, Phylogenetics, Microbial ecology

## Abstract

Active migration across semi-solid surfaces is important for bacterial success by facilitating colonization of unoccupied niches and is often associated with altered virulence and antibiotic resistance profiles. We isolated an atmospheric contaminant, subsequently identified as a new strain of *Bacillus mobilis*, which showed a unique, robust, rapid, and inducible filamentous surface motility. This flagella-independent migration was characterized by formation of elongated cells at the expanding edge and was induced when cells were inoculated onto lawns of metabolically inactive *Campylobacter jejuni* cells, autoclaved bacterial biomass, adsorbed milk, and adsorbed blood atop hard agar plates. Phosphatidylcholine (PC), bacterial membrane components, and sterile human fecal extracts were also sufficient to induce filamentous expansion. Screening of eight other *Bacillus* spp. showed that filamentous motility was conserved amongst *B. cereus* group species to varying degrees. RNA-Seq of elongated expanding cells collected from adsorbed milk and PC lawns versus control rod-shaped cells revealed dysregulation of genes involved in metabolism and membrane transport, sporulation, quorum sensing, antibiotic synthesis, and virulence (e.g., *hblA/B/C/D* and *plcR*). These findings characterize the robustness and ecological significance of filamentous surface motility in *B. cereus* group species and lay the foundation for understanding the biological role it may play during environment and host colonization.

## Introduction

Bacterial surface migration is increasingly recognized as an important aspect of bacterial fitness, allowing cells to sense and occupy new niches, translocate rapidly across surfaces, resist antibiotics and other deleterious circumstances, and shift virulence states [1–5]. Multiple forms of surface migration have been identified and are categorized based on the mechanism of motility, including swarming, surfing, gliding, twitching, and sliding [6]. Swarming requires flagella to propel bacteria in a concerted fashion in tendrils or waves on the surface of semi-soft (0.4–1%) and/or hard (1.5–3%) agar plates, with bacteria often displaying elongated and hyperflagellated cells at the motility front [2, 7]. Surfing also requires flagella (but not fimbriae) and occurs in the presence of the glycoprotein mucin and similar lubricant/wetting agents [5]. Twitching uses extension and retraction of type IV pili to pull cells forward [8]. Gliding occurs via a variety of active intracellular and membrane machinery to push cells across a surface [9]. Sliding is a passive spreading motion requiring surface-active substances, such as surfactants, exopolysaccharides, or membrane glycolipids, to lower surface tension [6, 9, 10]. Bacteria can often use multiple forms of surface motility depending on the environmental conditions.

*Bacillus* spp. are a large group of ecologically diverse rod-shaped spore-forming bacteria commonly found in soil, water, food, milk, air, animals, and are often isolated from humans [11–13]. Most *Bacillus* spp. are not associated with illness, and some species (e.g., *B. subtilis* and *B. licheniformis*) are considered potential probiotics [14]. However, many members of the *B. cereus* group, also known as *B. cereus sensu lato* (*s.l*.), are human pathogens. Currently, the *B. cereus s.l*. group contains more than a dozen species that are considered pathogens in humans including *B. cereus sensu stricto (s.s.), B. anthracis*, and *B. cytotoxicus*, and *B. thuringiensis* [15–17]. Some *B. cereus s.l*. species are used as probiotics in animals or humans or as food additives; however, there have been some safety concerns raised regarding encoded toxins [18–20]. *B. cereus s.s*. is among the best studied in the *B. cereus s.l*. group and is known to cause a wide array of diseases including gastrointestinal and respiratory system illnesses, endophthalmitis, bacteremia, central nervous system infections, and open wound infections [15]. *B. cereus s.s*. is most often reported to swarm on semi-soft agar by differentiation into hyperflagellated and elongated swarmer cells. Swarming versus non-swarming *B. cereus s.s*. produce higher levels of virulence factors, such as hemolysins, and have increased pathogenic potential by promoting access to new sites of infection [4, 15].

Our understanding of surface motility is largely limited to studies of single species swarming on semi-soft (<1.0%) agar cultured under specific temperature, nutrient, viscosity, and atmospheric conditions. Only a few types of bacteria, such as *Proteus, Vibrio*, and *Flavobacterium*, are known to demonstrate surface motility on surfaces other than semi-soft agar: *Proteus* and *Vibrio* can swarm on harder (1.5–3.0%) agar surfaces [21, 22], while *Flavobacterium johnsoniae* can glide on wetted glass surfaces [23]. However, ecological niches capable of supporting cell growth are highly complex and variable in their moisture content, atmospheric makeup, and nutrient composition, with microbes in this milieu also interfacing with a complex mixture of other microbes, debris, and metabolic products. The relatively few studies of multispecies swarms have yielded a rich source of information on the benefits of swarming to bacterial survival in mixed cultures, such as the ability to resist antibiotics, metabolic cross-feeding, as well as other bet hedging strategies [3].

Here, we isolated an atmospheric laboratory environment contaminant, subsequently identified as a new strain of *B. mobilis*, a member of the *B. cereus s.l*. group, which demonstrated significant cytotoxicity to HeLa and Caco-2 cells and showed robust filamentous motility when in direct contact with inactive *Campylobacter jejuni* cells, other bacterial biomass, and dried layers of milk and blood on hard (up to 5%) agar plates. Filamentous surface motility was conserved amongst representatives of other pathogenic *B. cereus s.l*. species and could be induced by phospholipids directly. Motile cells showed dysregulation of genes related to metabolism, sporulation, and virulence. The wide availability of phospholipid-containing compounds in natural niches occupied by *B. cereus s.l*., together with the conserved nature of this motility, suggests that filamentous motility is important for the ecological success of *B. cereus s.l*. in the environment and host.

## Materials and methods

### Strains and growth conditions

*Bacillus, Campylobacter*, and *Escherichia* species and strains used in this study are detailed in Supplementary Table S1. All bacteria were cultured in MH (Oxoid) broth or agar (MH with 1.5% w/v Difco Bacto agar) unless otherwise stated. Prior to each experiment, *Bacillus* spp. and *E. coli* were incubated aerobically at 30 °C overnight on 1.5% agar MH plates, or 2–4 h in MH broth culture under rotation. For selection of *B. cereus* 407 *Δfla* and *ΔmotA* strains 50 μg/mL kanamycin was added to plate and broth cultures. *C. jejuni* was cultured overnight on plates incubated microaerobically at 38 °C in a Sanyo tri-gas incubator (12% CO_2_ and 6% O_2_ in N_2_), or in broth cultures incubated shaking (200 rpm) in airtight containers with the Oxoid CampyGen System at 38 °C.

### Isolation, sequencing, and identification of *B. mobilis* ML-A2C4

The contaminant colony was re-streaked onto a fresh MH plate, and individual colonies were plated and stored frozen in 30% glycerol with MH. Genomic DNA for PCR assays was prepared by incubating a loopful of cells collected from an overnight plate in 5 mg/mL lysozyme in 10 mM Tris pH 8 at 37 °C overnight, bead beating cells for 1 min, then purifying using the Qiagen DNA extraction kit. Sequencing of 16S rRNA and *panC* were performed with primers listed in Supplementary Table S1 using iProof DNA polymerase (Bio-Rad), and BLASTn was used to identify sequence homology. Whole-genome sequencing was performed using the Illumina MiSeq and Pacific Biosciences (PacBio) RSII platforms as detailed in Supplementary Methods S1. Phylogenetic analysis and genotyping of ML-A2C4 used these whole-genome data compared with representative isolates of 18 putative and 3 effective *B. cereus s.l*. species listed in Supplementary Table S2 and detailed in Supplementary Methods S1. Virulence and antimicrobial resistance genes were detected with BTyper version 2.3.1 using a method described by Carroll et al. [24, 25] (Supplementary Table S3). Whole-genome sequence data for *B. mobilis* ML-A2C4 were deposited in GenBank under NCBI reference sequence CP031443 and assembly accession GCA_003612955.1. The protein-, rRNA-, and tRNA-coding genes of the genome were annotated using the NCBI Prokaryotic Genome Annotation Pipeline.

### Cytotoxicity

Bacterial supernatants of *B. mobilis* ML-A2C4 and type strains of *B. cereus* ATCC 14579 ^T^, *B. wiedmannii* FSL W8-0169 ^T^, *B. pseudomycoides* DSM 12442, and *B. mobilis* 0711P9-1 ^T^ were assessed in a HeLa and Caco-2 cell cytotoxicity assays [26] as detailed in Supplementary Methods S1. A total of 6–12 replicates were conducted per cell line for each bacterial supernatant.

### Plate set-up for filamentous surface motility assessment

Filamentous motility was assayed by spreading or spotting a substrate onto the surface of plates prepared using various nutrient types (MH, tryptone broth (TrB) (10 g/L tryptone, 5 g/L NaCl), Luria broth (LB), or brain heart infusion (BHI)) solidified at various agar concentrations (0.4–5% w/v) as indicated (refer to Supplementary Table S1 for all materials and conditions). Surface-adsorbed substrates tested included *C. jejuni* cells, other heat-killed cells, various milk types, defibrinated whole sheep blood, phosphatidylcholine (PC), human fecal extracts, and *E. coli* and *C. jejuni* inner and outer membranes (refer to Supplementary Table S1 for a complete list of substrates used). Control plates were minus added substrate. Substrate preparation and plating procedures are detailed in Supplementary Text S1. At the start of each motility experiment, 1 μL of 0.02 OD_600_ log phase cell culture was inoculated onto either the center of plates or next to (touching) patched lawns. Plates were incubated aerobically at the room temperature, 30, 37, or 42 °C, or in airtight containers microaerobically using the Oxoid CampyGen (~5% O_2_ and 10% CO_2_) or anaerobically using the Oxoid AnaeroGen (~<1% O_2_ and 9–13% CO_2_) atmosphere generation systems where indicated. Control and filamentous colony growth was measured as the longest visible growth distance either edge to edge for whole plate lawns, or from the point of inoculation to the edge of the visible growth for lawns that were spotted onto the plate. Plate photos were taken using the ChemiDoc^TM^ Touch Imaging System (Bio-Rad) with 1–3 s exposure, and images were processed for optimal brightness, contrast, and visual clarity using Adobe Photoshop Lightroom CC (2015. 10 Release).

### Microscopy

Sterile glass slides were placed at the bottom of individual plates before nutrient agar was poured and solidified. Agar plates were inoculated with substrates and cells as described above. To isolate the growth region of interest for microscopy the embedded glass slides were cut out of the agar, ~1.5 by 1.5 cm squares of agar atop the extracted slides containing the area of interest were isolated (excess agar on the slides were discarded), and topped with coverslips. Imaging was performed using a DIC inverted microscope (Nikon eclipse TE2000-U) using oil immersion within 1 h of sample processing. Images were processed for optimal brightness, contrast, and visual clarity using Adobe Photoshop Lightroom CC.

### RNA sequencing

Control, 10% skim milk, and 2% PC lawn plates were prepared and inoculated with ML-A2C4 as described above. Three independent biological replicates were sampled for each of the three growth conditions (nine replicates total). For each replicate, cells from two to four plates were scraped from the outermost edges (1–2 mm) of the control or filamentous colonies at 24 h post inoculation directly into RNA Protect Bacteria Reagent (QIAGEN). Samples were processed and sequenced using Illumina HiSeq 2500 as described in Supplementary Methods S1. Significantly differentially expressed genes (false discovery rate ≤0.05 and fold change ≥±2) were identified using DESEQ2 1.20.0 and R. Refer to Supplementary Methods S1 for additional methods used for RNA-Seq data analysis. RNA-Seq data for this study were submitted to the NCBI Gene Expression Omnibus database under study GSE136873 using accession numbers GSM4060077/8/9 for control-plated replicates, GSM4060080/1/2 for filamentous colonies growing on milk lawns, and GSM4060083/4/5 for filamentous colonies growing on PC lawns.

### Statistics

Statistical analyses of bacterial growth were analyzed and graphed using Graphpad Prism 7, and statistical differences calculated using the Student’s *t* test with Welch’s correction. Statistical analysis of colony diameter over time (e.g., 48 vs. 24 h) used repeated measures one-way ANOVA with Greenhouse–Geisser correction. For cytotoxicity testing, a Welch’s test and the Games–Howell post-hoc test were used to calculate the statistical differences using R version 3.4.3, and a Bonferroni correction was applied to correct for multiple comparisons.

## Results and discussion

### Isolation of an environmental contaminant with preferential expansion on *C. jejuni* cell lawns

**Fig. 1:**
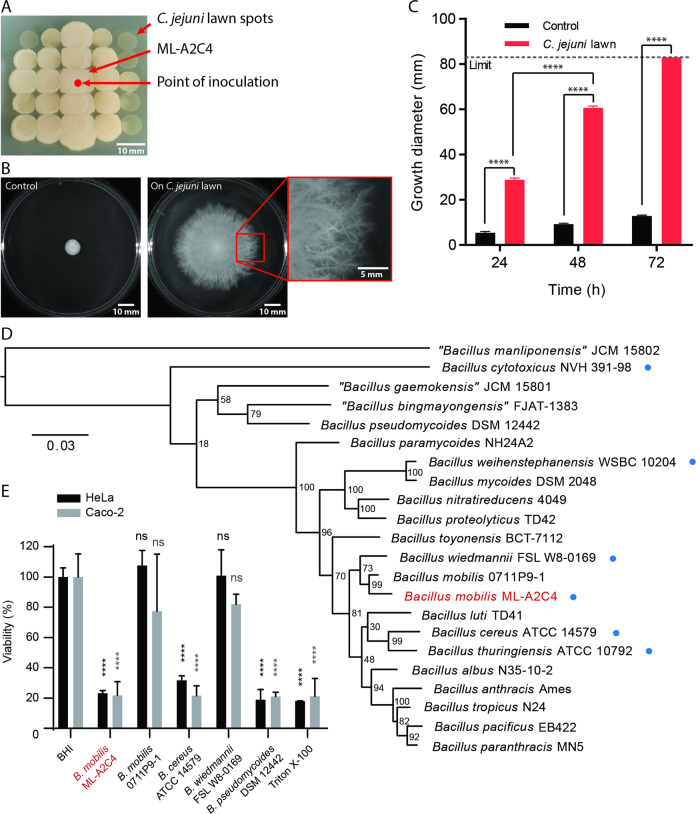
Identification of the filamentous motile environmental isolate as *Bacillus mobilis* ML-A2C4. **a** ML-A2C4 filamentous growth on *C. jejuni* lawn spots (small circles). **b** ML-A2C4 growth on a control 1.5% agar MH plate (left) and on a MH plate spread with a full confluent *C. jejuni* lawn (center) after 48 h aerobic incubation at 30 °C. The red box shows a close-up view of the filaments at the growth edge (right). **c** Quantification of the visible growth diameter on control MH plates (black bars) and plates with *C. jejuni* lawns (red bars) over time (*n* = 5) with error bars indicating standard deviation (SD). Statistical analysis was performed for growth diameter on *C. jejuni* lawn plates versus control plates using the Student’s *t* test with Welch’s correction, and for 48 vs. 24 h using repeated measures one-way ANOVA, with ****p < 0.0001. The limit (dotted line) represents the diameter of the MH plate. **d** Phylogenetic placement of ML-A2C4 (red type) based on the core genome SNP comparison with other members of the *B. cereus* group. Blue dots indicate additional *B. cereus* species selected for comparative phenotypic testing. **e** Cytotoxicity *of B. cereus* ATCC 14579, *B. mobilis* 0711P9-1, *B. pseudomycoides* DSM 12442, *B. wiedmannii* FSL W8-0169, and *B. mobilis* ML-A2C4 supernatants on HeLa (black) and Caco-2 (gray) cells relative to negative control (EMEM + FBS supplemented with BHI medium). Triton X-100 (0.05%) was included as a positive cytotoxicity control. ****p < 0.0001 indicates that there is significant difference in the cytotoxicity between the bacterial supernatants and the respective BHI negative control for each cell line as determined using the Games–Howell test.

We observed a contaminant colony that paradoxically grew preferentially on small, spot-plated lawns of *C. jejuni* cells on Mueller Hinton (MH) agar (1.5% w/v). The MH plate had previously been inoculated with *C. jejuni* cells spotted and incubated microaerobically at 38 °C overnight before being stored for several days aerobically at room temperature. Transfer of contaminant cells onto new, similarly prepared spot-plated lawns of *C. jejuni* resulted in the contaminant again growing preferentially atop the *C. jejuni* lawns, with minimal growth on the rich agar in between spots of *C. jejuni* lawns (Fig. 1a). The contaminant was isolated for further study and the strain named ML-A2C4.

A two day spread plating technique was developed to achieve full, dense, and confluent lawns of *C. jejuni* (“*C. jejuni* lawn” plates; refer to Supplementary Methods S1), which were stored aerobically at room temperature for up to 5 days. Control conditions were comparable plates without *C. jejuni*. ML-A2C4 was inoculated at the center of each plate, and expanded rapidly on those spread with *C. jejuni* lawns but not on control plates (Fig. 1b). The expanding ML-A2C4 colony was flat and often showed a dense white inner ring with a less dense outer ring. The outer ring had fine tendrils that exhibited a distinctive filamentous pattern radiating outward in random directions at the edges (Fig. 1b). When incubated aerobically at 30 °C, ML-A2C4 expanded rapidly on *C. jejuni* cell lawns at ~32 mm/day, filling the entire plate (83 mm diameter) between 48 and 72 h post inoculation (Fig. 1c). In contrast, colonies on control plates grew modestly over time (~4 mm/day) and did not exhibit filamentous edges (Fig. 1b, c). Further investigation into this strain and the filamentous surface migration phenomenon was undertaken as bacterial motility on hard agar is relatively rare, surface motility dependence on the presence of other cell lawns had not yet been reported, and the distinctive filamentous expansion of the colony appeared unique.

### The contaminant was identified as a new *B. mobilis* strain

We hypothesized that ML-A2C4 originated from the air since the plate on which it was originally isolated had been dried with the lid off prior to use. Light microscopy of cells from control plates showed that they were rod-shaped spore formers (data not shown). Initial 16S rRNA sequencing revealed that ML-A2C4 belonged to the *B. cereus* group, and *panC* sequencing using the 7-clade *panC* classification scheme demonstrated that it belonged in clade II [13]. Therefore, we performed whole-genome sequencing of ML-A2C4 using both PacBio and Illumina MiSeq and compared it with the other members of the *B. cereus s.l*. species. The genome of ML-A2C4 was 5.47 Mb with an average GC content of 35.6%. Core genome comparative analyses with 18 valid and three effective *B. cereus s.l*. species type strains revealed a close relatedness to the *B. mobilis* type strain 0711P9-1 based on 1142 core SNPs identified using kSNP3 [27] (Fig. 1d and Supplementary Table S2). The genome of *B. mobilis* ML-A2C4 was uploaded into Genbank as NCBI reference sequence NZ_CP031443.1 and represents the first complete annotated genome for *B. mobilis*.

ML-A2C4 possesses 23 known or putative virulence genes and five antimicrobial resistance genes (Supplementary Table S3), including the *hblABCD* and *nheABC* genes encoding the well-characterized diarrheal enterotoxin hemolysin BL and nonhemolytic enterotoxin Nhe [28–31]. To further characterize the pathogenic potential of this strain, we evaluated HeLa and Caco-2 cell intoxication using bacterial supernatants (Fig. 1e). Survival was determined based on the metabolic conversion of WST-1 into a colored dye. ML-A2C4 supernatant reduced HeLa and Caco-2 cell viability to 23% and 22% of untreated, respectively. This was comparable with the cytotoxic-positive control *B. cereus* ATCC 14579 supernatant, which reduced HeLa and Caco-2 cell viability to 32% and 22% of untreated, respectively, and *B. pseudomycoides* DSM 12442 (19% viability for HeLa and 21% for Caco-2 cells). The *B. mobilis* reference strain 0711P9-1 and *B. wiedmannii* FSL W8-0169 did not demonstrate cytotoxicity in either HeLa or Caco-2 cells. The pronounced cytotoxicity of ML-A2C4 may have implications for pathogenesis of certain *B. mobilis* strains.

### Filamentous motility was robust under a wide range of environmental conditions

*B. mobilis* is phylogenetically closely related to *B. cereus s.s*. (Fig. 1d), which swarms by elongation and hyper-flagellation of individual cells when inoculated on the surface of semi-soft (0.4–1.0%) agar [4, 32]. ML-A2C4 inoculated onto similar swarm plates (LB and TrB with 0.5 and 0.7% agar) failed to swarm (data not shown). A *plcR* deletion mutant of *B. cereus s.s*. also demonstrates dendritic colony morphology, which is thought to occur as a result of a sliding action of the filamentous cells after 96 h incubation on 0.7% agar made with low-nutrient defined media [33]. In contrast, the filamentous growth of ML-A2C4 on *C. jejuni* lawns occurred much earlier (before 24 h), was morphologically distinct, was robust even on rich MH agar plates at a much higher agar concentration, had a faster expansion rate, and was inducible. Therefore, we concluded that the filamentous growth of ML-A2C4 was distinct from the previously reported swarming and dendritic sliding phenotypes of *B. cereus s.s*.

**Fig. 2:**
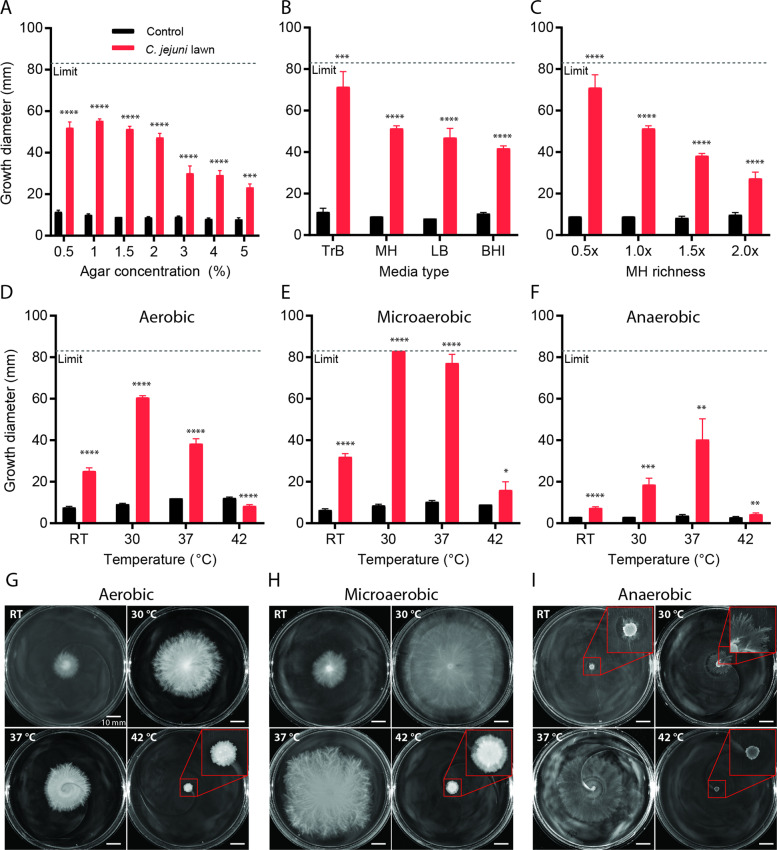
Robustness of ML-A2C4 surface motility under different conditions. Impact of agar concentration (**a**), media type (**b**), MH richness (**c**), temperature (**d**–**i**), aerobic (**d**, **g**), microaerobic (**e**, **h**), and anaerobic (**f**, **i**) conditions on ML-A2C4 growth after 48 h incubation on control plates (black bars) and plates spread with *C. jejuni* lawns (red bars). The measurement limit represents the diameter of the plates. Testing was performed at *n* = 5–8 per condition. Pictures show representative plates. Red boxes show greater magnification of the indicated area. Statistical analysis was performed for growth diameter on *C. jejuni* lawn plates versus control plates where indicated using the Student’s *t* test with Welch’s correction. *p* values are represented as: ****p < 0.0001, ***p < 0.001, **p < 0.01, *p < 0.05.

We assessed ML-A2C4 filamentous growth under a wide range of conditions using *C. jejuni* lawn plates prepared with different agar concentrations, nutrient types, nutrient richness, and incubated at different temperatures and oxygen concentrations. No notable variability was observed in the *C. jejuni* lawns on these plates prior to ML-A2C4 inoculation. Control conditions were the corresponding plates without *C. jejuni*. ML-A2C4 filamentous motility occurred on *C. jejuni* lawn plates at all agar concentrations tested (0.5–5% w/v); however, it was fastest when plate agar concentrations were less than 2% (Fig. 2a). Motility of ML-A2C4 on *C. jejuni* lawn plates containing up to 5% agar was unexpected, since even the robust swarmer *P. mirabilis* exhibits minimal motility on 3% agar and does not swarm at all on 4% agar [22, 34], while other swarming cells like *P. aeruginosa* are inhibited at 0.6% agar [35]. ML-A2C4 showed filamentous expansion on *C. jejuni* lawns atop 1.5% agar plates made with different nutrient types including TrB, MH, LB, and BHI; however, the expansion rates appeared inversely correlated with nutrient richness and were faster on plates with less nutrient rich media (e.g., TrB, 15 g/L solids) versus highly rich media (e.g., BHI, 37 g/L solids) (Fig. 2b). To test different nutrient richness levels using the same medium, we made plates containing 0.5× and up to 2× the amount of MH powder normally used prior to inoculation of *C. jejuni* lawns. ML-A2C4 exhibited filamentous motility under all MH conditions tested; however, expansion was fastest when the lawn plates contained lower levels of MH (Fig. 2c). ML-A2C4 growth on control plates without *C. jejuni* lawns was comparable for all agar concentrations, media types, and MH concentrations (Fig. 2a–c). Inoculation of ML-A2C4 onto *C. jejuni* lawn plates and subsequent incubation at multiple temperatures (22–42 °C) and oxygen levels (aerobic, microaerobic, and anaerobic conditions) demonstrated that cell growth and filamentous motility occurred over a wide range of temperature and oxygen conditions but was most rapid under microaerobic incubation at 30 °C (Fig. 2d–i). *Bacillus* spp. are facultative anaerobes, but fermentative growth is much less effective than aerobic growth [36]. As expected, ML-A2C4 growth on both control and *C. jejuni* lawn plates was inhibited when incubated under anaerobic conditions (Fig. 2f, i). However, filamentous expansion was still evident, especially at 37 °C, despite lack of accumulated cell mass (Fig. 2i). For all the experiments described below, ML-A2C4 filamentous surface motility was evaluated using 1.5% MH agar plates incubated aerobically at 30 °C unless otherwise stated.

### Filamentous motility was induced by other metabolically inactive bacterial cells, blood, and milk

**Fig. 3:**
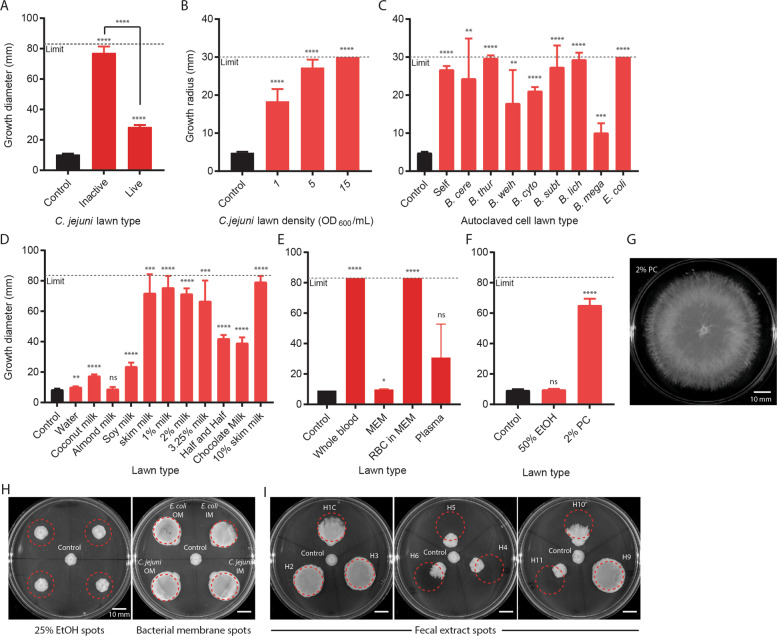
Conditions that induced filamentous expansion. **a** Comparison of inactive (pre-prepared plates) and live (fresh plates) *C. jejuni* cell lawns that induced ML-A2C4 spreading after microaerobic incubation at 38 °C (*n* = 5). **b** ML-A2C4 expansion on adsorbed *C. jejuni* cell lawns prepared by autoclaving cells at various densities (*n* = 8). The measurement limit is lower because the cell lawns were applied onto MH plates as small ~30 mm diameter spots. ML-A2C4 was inoculated at the edge of the lawn. **c** ML-A2C4 expansion on 15 OD_600_/mL autoclaved cell lawns of multiple cell types including itself (“self”) (*n* = 8). Shortened species names listed in order are: *B. cereus, B. thuringiensis, B. weihenstephanensis, B. cytotoxicus, B. subtilis, B. licheniformis, B. megaterium*, and *E. coli*. ML-A2C4 expansion on multiple types of milk products (**d**), blood products (**e**), and phosphatidylcholine (PC; **f**, **g**) (*n* = 5). MEM minimal essential medium, RBC red blood cell, EtOH ethanol. **h** Representative plates from *n* = 3 testing of ML-A2C4 growth on 25% EtOH spots (left) and *E. coli* and *C. jejuni* inner membrane (IM) and outer membrane (OM) preparations. **i** Representative plates from *n* = 3 testing of ML-A2C4 growth on nine human fecal extracts (left: H1C, H2, and H3; center: H4, H5, and H6; right: H9, H10, and H11). Dotted lines in **h** and **i** indicate area where substrate was dried onto the plate. As a control, ML-A2C4 was spotted in the middle of plates shown in **h** and **i** where there was no substrate. All data shown were taken after 48 h incubation. Statistical analysis was performed for growth diameter on *C. jejuni* lawn plates versus control plates where indicated using the Student’s *t* test with Welch’s correction. *p* values are represented as: ****p < 0.0001, ***p < 0.001, **p < 0.01, *p < 0.05, ns not significant.

As noted above, *C. jejuni* lawn plates were prepared and stored aerobically at room temperature for up to 5 days before each experiment. *C. jejuni* is microaerophilic and thermophilic and does not grow aerobically or at room temperature, thus the cells on these plates are likely metabolically inactive and/or dead [37]. To determine if ML-A2C4 could expand on *C. jejuni* lawns containing actively growing cells, we inoculated freshly grown *C. jejuni* plates with ML-A2C4 and incubated them microaerobically at 38 °C, a condition under which most *C. jejuni* cells are expected to remain viable. Filamentous expansion on live *C. jejuni* cell lawns was reduced versus on plates containing the inactive lawns (Fig. 3a). ML-A2C4 filamentous growth was also inhibited when inoculated next to or on top of live lawns of other bacteria such as *E. coli* (data not shown), suggesting that either the compound(s) triggering filamentous motility were not accessible in living bacteria, or that living bacteria were capable of inhibiting ML-A2C4. Lawns of autoclaved *C. jejuni* spotted and dried onto agar plates induced filamentous growth in a concentration-dependent manner (Fig. 3b), and ML-A2C4 showed filamentous motility on heat-killed lawns of all bacterial cell types tested, including itself (Fig. 3c).

A variety of additional compounds, selected to represent different nutrients, cellular products, stressors, host factors, and other environmental factors that *Bacillus* might encounter, were screened to determine if there were additional triggers. Filamentous motility was not induced when plates were inoculated with protein derivatives, nucleotides, sugars, high levels of nutrients, or various other chemicals (see Supplementary Table S1 for a full list of tested substrates and concentrations). However, filamentous motility was very robust on plates containing adsorbed lawns of milk and blood (Fig. 3d, e). These findings were particularly notable since *Bacillus* spp. are commonly isolated from contaminated milk and have been shown to cause bacteremia in humans [15]. Filamentous expansion was robustly induced on lawns of commercial cow-milk products ranging from skim to half-and-half, and was modestly induced with soy milk but not coconut or almond milk (Fig. 3d). We separated whole blood into red blood cells [RBCs; re-suspended in minimal essential medium (MEM)] and plasma by centrifugation. RBCs induced filamentous growth comparable with whole blood, but filamentous expansion was reduced and inconsistent in plasma (Fig. 3e), while fetal bovine serum likewise did not induce filamentous growth (data not shown). Direct contact also appeared to be required for initiation of filamentous growth, since ML-A2C4 did not move in the direction of tested compounds when inoculated near (but not touching) spots of *C. jejuni* cell lawns, blood, or milk, and did not expand when inoculated on top of 5% or 15% sheep blood agar where the RBCs were embedded within the agar plate (data not shown).

### Filamentous motility was induced by phospholipids and human fecal extracts

Phospholipids are present in membranes of bacterial cells and RBCs, and soluble phospholipids are found in cow milk and soy milk [38–42]; therefore, we suspected that phospholipids might be an inducer. PC was utilized as a representative phospholipid for initial testing. Filamentous growth readily occurred on plates spread with PC (0.5 mL of 2% w/v in 50% EtOH) (Fig. 3f, g); EtOH did not impact ML-A2C4 growth (Fig. 3f). Filamentous growth also occurred readily on extracted inner and outer membrane fractions from *E. coli* and *C. jejuni*, which are composed of primarily of phosphatidylglycerol and phosphatidylethanolamine [40, 43], suggesting that filamentous growth could be induced by multiple phospholipid types (Fig. 3h).

Since filamentous expansion was optimally induced under low nutrient, moist, microaerobic conditions between 30 and 37 °C while in contact with dead bacterial cells and/or phospholipids, and as *B. cereus s.l*. organisms are often gut colonizers, we hypothesized that this filamentous motility might occur in the intestinal milieu. While the lumen of the gut can be largely anaerobic, the oxygen content near the intestinal epithelia is better described as microaerobic, and aerotolerant microbes are well suited to thrive in this ecological niche [44]. To determine if human gut contents could induce filamentous motility, nine sterile human fecal extracts collected and purified from a separate study were spotted onto agar plates [45]. ML-A2C4 exhibited filamentous expansion when inoculated on five out of nine human fecal extracts (Fig. 3i). This indicated that human gut contents could selectively induce filamentous motility. As filamentous expansion was not induced with all human fecal extract samples, we hypothesize that other factors impacting extract composition such as person to person differences, diet, stool frequency, and variability during extract preparation also impacted ML-A2C4 motility [45, 46].

### Filamentous motility was conserved to varying degrees amongst members of the *B. cereus s.l*. group

**Fig. 4:**
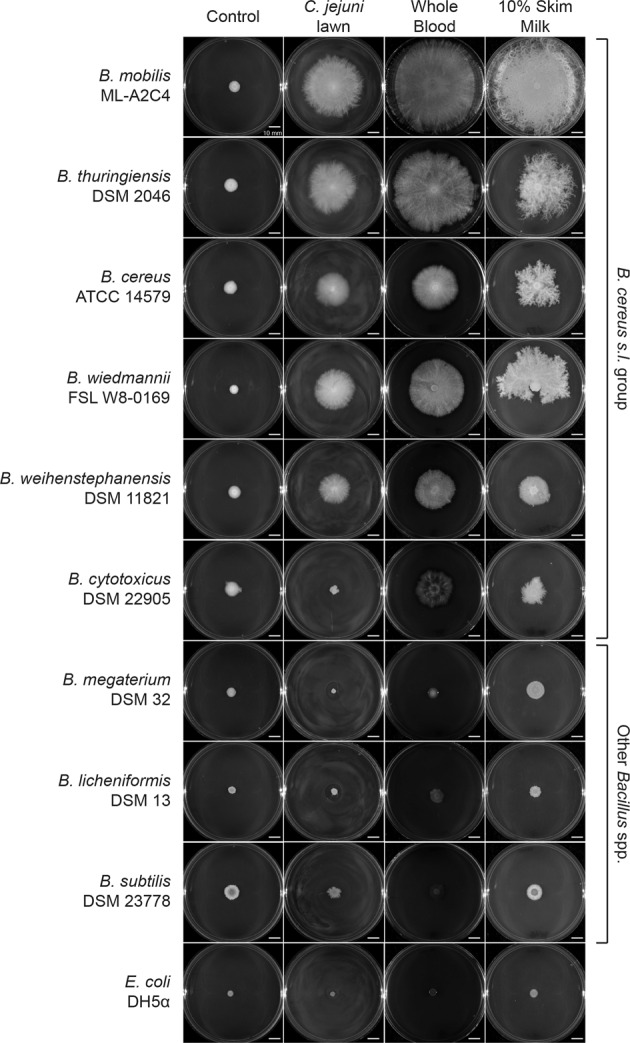
Growth and expansion of *Bacillus* spp. and *E. coli.* Growth after 48 h on control 1.5% agar MH plates (left column), plates spread with *C. jejuni* lawns (middle left column), whole blood (middle right column), and 10% skim milk (right column) preparations. Testing was performed at *n* = 5, representative images are shown.

To determine if filamentous motility was conserved amongst other *Bacillus* spp., reference strains of eight different species distributed across the *Bacillus* genus were screened. These included five from the *B. cereus s.l*. group (*B. cereus s.s*. ATCC 14579, *B. thuringiensis* DSM 2046, *B. wiedmannii* FSL W8-0169, *B. weihenstephanensis* DSM 11821, and *B. cytotoxicus* DSM 22905), and three others including *B. subtilis* DSM 23778, *B. licheniformis* DSM 13, and *B. megaterium* DSM 32. *B. subtilis* DSM 23778 does not produce surfactin, but *B. licheniformis* DSM 13 does [47, 48]. *E. coli* DH5α was used as a negative control. Filamentous motility on *C. jejuni* cell lawns, 10% skim milk, and whole blood adsorbed onto 1.5% MH agar plates was observed for four out of five *B. cereus* species (Fig. 4). *B. cytotoxicus* displayed expansion on blood and milk but not on *C. jejuni* cell lawns (Fig. 4). *B. cytotoxicus* is the most phylogenetically distant member of the *B. cereus s.l*. group tested (Fig. 1d), so while filamentous growth appeared generally conserved, different members of the group could have different growth preferences. Filamentous expansion was not observed with *B. subtilis, B. megaterium, B. licheniformis*, or *E. coli* when incubated on lawns of *C. jejuni* cells, blood or milk (Fig. 4), indicating that filamentous surface motility is partially conserved in the *B. cereus s.l*. organisms but not in other environmental *Bacillus* species.

### Microscopy revealed chains of elongated cells at expanding edge

**Fig. 5:**
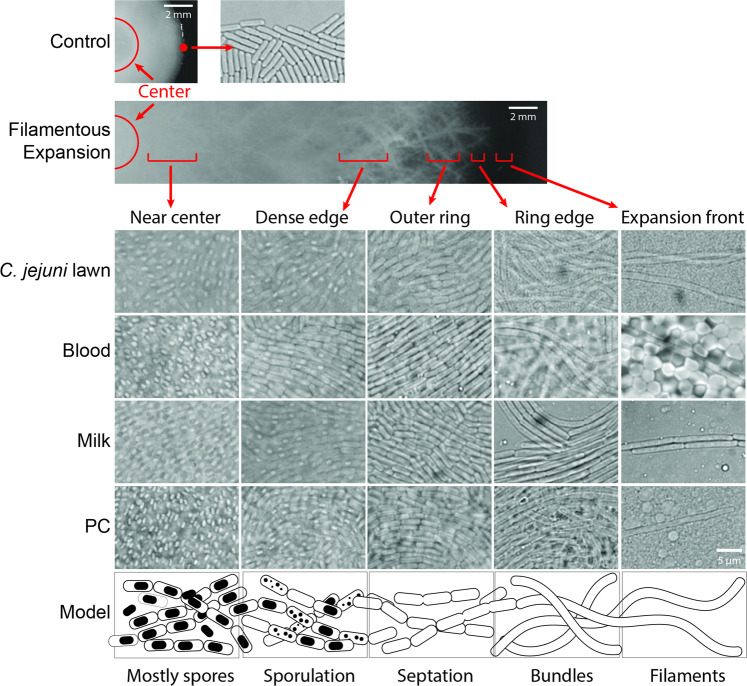
Microscopy of ML-A2C4 cells on control plates and plates spread with *C. jejuni* lawns, whole blood, 10% skim milk, and PC. Representative images are shown. Five distinct stages of cell and colony morphology were observed from the visible colony edge to the center of the plate. A model of the growth phenotypes was created based on these visual observations to highlight various stages of filamentous spreading.

Under light microscopy, filamentously expanding colonies of ML-A2C4 on plates with *C. jejuni* cell lawns, milk, blood, and PC showed that edges of the expanding circle were populated by very long chains of cells with minimal visible septation and/or cell division (Fig. 5 “Expansion front” column), whereas the edges of colonies on control plates were mostly populated by a single layer of rod-shaped individual cells. The elongated filamentous chains appeared oriented in all directions and undulated through multiple focal planes of the *C. jejuni* or RBC cell lawns versus resting on top of the cell layers or between the cells and the agar, suggesting that they might be burrowing through the cell layers. The tips of the elongated cells also appeared to extend over time with a smooth continuous forward motion, which we hypothesize was driven by growth and extension of the long filament. At the visible edge of the filamentous colony, the cell population was more dense and appeared as bundles of elongated cell chains (Fig. 5 “Ring edge” column), and in the first few millimeters of the growth ring the cells had distinct septa (“Outer ring” column). The denser, inner circle of cell growth was populated with a high proportion of sporulating cells (Fig. 5 “Dense edge” column), and the population near the center of the colony largely consisted of spores (Fig. 5 “Near center” column).

Comparable with ML-A2C4, the expanding fronts of the five other *B. cereus s.l*. species displaying the above-described filamentous motility also exhibited elongated cells when grown on lawns of *C. jejuni*, milk, and blood (Supplementary Fig. S1), with the exception of *B. cytotoxicus* which, as noted above, did not expand on *C. jejuni* cell lawns. We also examined the morphology of *B. subtilis, B. licheniformis*, and *B. megaterium* that did not exhibit filamentous motility. Unexpectedly, *B. subtilis* cells grown on *C. jejuni* and blood lawns showed elongated cells comparable with those observed for the *B. cereus s.l*. organisms, even though there was no characteristic colony expansion observed on those plates (Supplementary Fig. S1). *B. licheniformis, B. megaterium*, and *E. coli* cell morphology remained comparable with the control situation under all growth conditions.

### Motile cells exhibited significant gene transcription differences compared with control cells

**Fig. 6:**
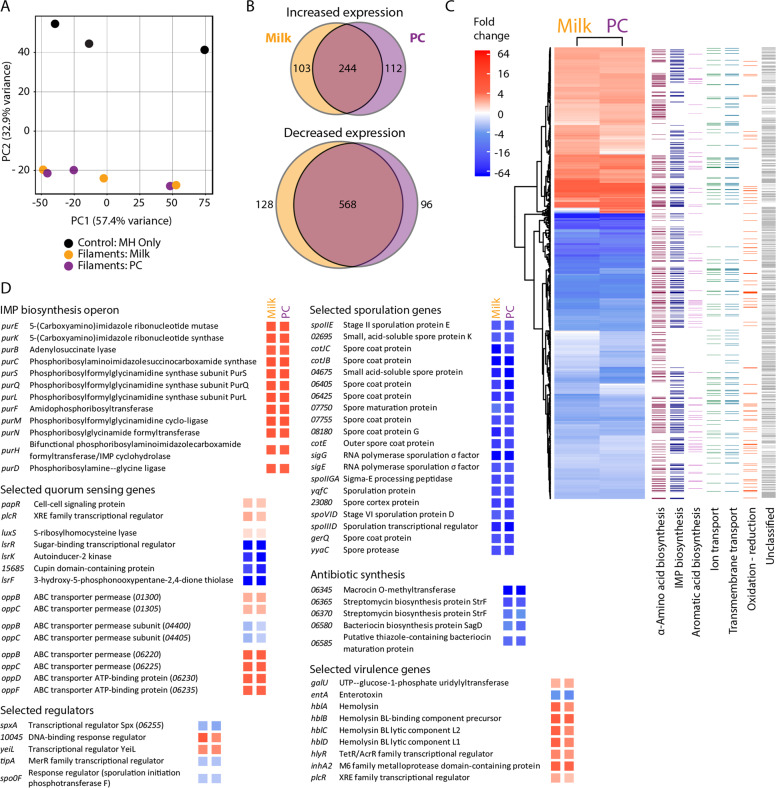
Differential expression in filamentous motility expanding cf. control colonies. **a** Principle component analysis of control cells (black), filamentous cells on 10% skim milk lawns (orange) and on PC lawns (purple). **b** Venn diagrams of genes that showed increased (top) and decreased (bottom) transcription during filamentous expansion in comparison with control cells. **c** Heat map of all differently transcribed genes during filamentous growth in milk and PC (wide columns) and the gene ontology (GO) categories assigned to those genes (thin columns). **d** Lists of differently transcribed genes organized based on predicted function with the fold change represented as a colored box corresponding to the fold change scale in the heat map.

We performed RNA-Seq transcriptomic analysis using the Illumina HiSeq 2500 system on the highly motile cells from milk and PC plates to better understand putative genetic mechanisms associated with and driving filamentous growth. ML-A2C4 cells were collected from the edges (outermost 1–2 mm) of filamentous colonies on milk and PC lawn plates incubated aerobically at 30 °C for 24 h to isolate actively growing cells, and gene expression profiles were compared with those of cells collected from the edges of colonies on control MH agar plates without adsorbed milk or PC incubated under the same conditions. Principle component analysis showed a clear distinction between gene expression patterns of filamentous motility cells when compared with control cells (Fig. 6a). Filamentous cells harbored 456 genes with higher and 792 genes with lower transcription during growth on milk and/or PC, with the majority of the differentially transcribed genes common to both milk- and PC-induced growth conditions (Fig. 6b, c, Supplementary Table S4). Functional classification based on gene ontology (GO) categorization showed that filamentous motility cells differentially modulated transcription of genes involved in amino acid biosynthesis, de-novo inosine monophosphate (IMP) biosynthesis, aromatic acid biosynthesis, ion and transmembrane transport and oxidation–reduction processes (Fig. 6c, Supplementary Table S5). However, ~60% of genes could not be assigned a functional GO classification in part because this strain provided the first fully circular annotated genome for *B. mobilis*.

Genes exhibiting the highest dysregulation in filamentously motile versus control cells included several involved in IMP biosynthesis (including all 12 genes of the purine biosynthesis operon *purEKBCSQLFMNHD*) and virulence [including *hblA/B/C/D, hlyR*, *inhA2*, *galU* (homolog of *bpsE*, an exopolysaccharide gene), and *plcR*], which showed some of the largest increases in transcription, while genes involved in sporulation and antibiotic synthesis showed the greatest downregulation of transcription in filamentous cells (Fig. 6d). Reduced transcription of sporulation related genes, some having up to 75-fold lower transcription than in control cells, was consistent with microscopic observations that cells near the edge did not have spores (Fig. 5). At least 15 regulators were more highly expressed, and 12 had lower expression during growth on milk and PC lawns (Supplementary Table S6). Many of the regulators appeared related to sporulation (e.g., *spoIIID, spo0F*), virulence (e.g., *plcR*), antibiotic response (e.g., *tipA*), and metabolism (e.g., *spxA, yeiL, 10045*) (Fig. 6d). Genes likely involved in quorum sensing that were differentially transcribed in filamentous colonies included *plcR*-*papR* (1.7- to 2.2-fold higher), two out of three clusters of the *opp* oligopeptide transport system genes (*oppBCDF*; 2.0- to 5.2-fold higher), and autoinducer-2 (AI-2) genes (22- to 182-fold lower) (Fig. 6d, see Supplementary Table S6 for a full list) [49–51]. The reduction in genes responsible for AI-2 intracellular processing suggested that it might be involved in filamentous growth, but further assessment is required.

To determine if gene regulation changes we observed during filamentous motility on milk and PC were similar to differential gene expression during *B. cereus s.s*. swarming in semi-soft agar, we compared the microarray transcription profile of *B. cereus* ATCC 14579 swarming versus non-swarming conditions (published in Salvetti et al. [4]) to the RNA-Seq results obtained for ML-A2C4. ML-A2C4 possesses 86 of the reported 119 *B. cereus* genes showing more than a twofold difference in transcription during swarming (including hemolysin genes) (Supplementary Table S7). 21 of 86 genes (24%) showed expression patterns similar to that observed during *B. cereus* swarming [including hemolysin genes (*hblABC)*, fermentation and oxidative phosphorylation genes (*pflAB* and alcohol dehydrogenases), and membrane transporters and stress proteins], 25 (29%) had the opposite pattern of expression [including purine, histidine, and lysine biosynthesis genes (*purEBSLN*, *xpt*, *hisAFHL*, and *lysA*), and many hypothetical proteins], and 15 (17%) did not show differences in expression during filamentous growth. While swarming *B. cereus* cells showed a 4.0-fold higher expression of flagellin [4], no such increase was observed in filamentously growing ML-A2C4 on either milk or PC lawns. To further assess whether filamentous motility requires flagella, we specifically looked at the ML-A2C4 44-gene flagellar locus *08515* to *08735* involved in biosynthesis and control of the flagellar apparatus. Only the *cheY* response regulator showed >2-fold difference in transcription during filamentous growth in milk and PC (2.7- and 2.3-fold, respectively). To confirm flagella were not required for filamentous motility, we tested *B. cereus* 407 cells with deletions in *motA* and the *fla* locus for surface motility. The *Δfla* strain does not produce flagella, and both the *Δfla* and the *ΔmotA* strains are nonmotile in a semi-soft agar swim plates [52]. The wild-type, *ΔmotA*, and *Δfla* strains showed comparable filamentous motility on *C. jejuni* lawns, blood, and milk, which verified that filamentous motility likely does not require the flagellar apparatus (Supplementary Fig. S2). Thus, while many similar genes appear to be controlled in both swarming and our above-described filamentous growth, the overall regulation of these metabolic and motility systems, as well as the mechanism of motility, are unique.

## Conclusions

Here we characterized a novel inducible filamentous motility conserved among *B. cereus s.l*. species, which allowed cells to move through and colonize milieu such as bacterial debris, blood, and milk lawns on hard agar. We further gained some insight into mechanisms driving motility and showed that actively expanding cells were metabolically distinct, had increased expression of some virulence genes, and moved as chains of elongated cells which appeared to burrow through dense bacterial and blood cells. Phospholipids are abundant in the ecological niches where *B. cereus s.l*. organisms thrive, such as milk and the gut, and even soil environments with dead bacterial or animal debris. Filamentous motility was robust under a wide variety of conditions but fastest in a warm, moist, and microaerobic environment, and could be induced with PC alone. We do not yet know how phospholipids may induce this filamentous motility, or whether phospholipids alone can act directly as a surfactant and/or lubricant to promote surface motility. The human gut is warm, moist, oxygen depleted, and rich in phospholipids from a complex mixture of food breakdown products, the microbiome, mucus, bile components, and shed intestinal epithelial cells [46, 53–55]. The colonic mucosa can even have up to 20 times the concentration of phospholipids compared with the adjacent fecal material due in part to active transport of PC into the mucus lining [53, 56]. We hypothesize that in the intestinal environment, filamentous motility of *B. cereus s.l*. bacteria aids cells in burrowing through the intestinal lumen or the colonic mucosa and contributes to colonization and pathogenesis in the host. Microscopic ecological environments can be complex and diverse with heterogeneous microniches, and we hypothesize that this filamentous motility, along with previously described swimming, swarming, and sliding activity, promotes active occupation of *B. cereus s.l*. into any permissive niche. Future work will provide insight into what motors, if any, drive filamentous motility, how specifically phospholipids contribute to and/or modulate this motility, specific *B. cereus/mobilis* genes involved, and how this motility might contribute to bacterial fitness and ecological success in the environment and the host.

## Supplementary information

Supplemental Material Legends

Supplemental Table S1

Supplemental Table S2

Supplemental Table S3

Supplemental Table S4

Supplemental Table S5

Supplemental Table S6

Supplemental Table S7

Supplemental Methods S1

Supplemental Figure S1

Supplemental Figure S2
