# Supplementary material for: Unique inducible filamentous motility identified in pathogenic Bacillus cereus group species
Source: ISME J. 2020 Aug 7;14(12):2997–3010. doi: 10.1038/s41396-020-0728-x (PMC7784679; doi:10.1038/s41396-020-0728-x)
Supplement: Supplementary file 1 — Supplemental Material Legends [file 41396_2020_728_MOESM1_ESM.docx]

**SUPPLEMENTAL MATERIAL LEGENDS**

**Supplemental Table S1.** Bacterial species, primers, and reagents used.

**Supplemental Table S2.** *Bacillus* spp. used for phylogenetic analysis and placement of *B. mobilis* ML-A2C4.

**Supplemental Table S3.** Virulence and antimicrobial resistance gene homologs encoded by *B. mobilis* ML-A2C4.

**Supplemental Table S4.** RNA-Seq results of filamentous growth on milk and PC vs. control.

**Supplemental Table S5.** Gene ontology categories for differentially expressed genes.

**Supplemental Table S6.** List of quorum genes and regulators with differential expression for filamentous growth on milk and PC vs. control.

**Supplemental Table S7.** Comparison of microarray results for *B. cereus* ATCC 14579 swarming vs. non-swarming to RNA-Seq results for *B. mobilis* ML-A2C4 filamentous motility on milk and PC vs. control.

**Supplemental Methods S1.** Additional details of methods used.

**Supplemental Figure S1.** Microscopy of cells at the edge of the bacterial colonies grown on control 1.5% agar MH plates (left column), and MH plates spread with *C. jejuni* lawns (middle left column), blood (middle right column), and 10% skim milk (right column).

**Supplemental Figure S2.** Colony growth of *B. cereus* 407 *ΔmotA* and *Δfla* locus deletion strains compared to wildtype (WT) strain and ML-A2C4 under filamentous growth-inducing conditions.
