# Supplementary material for: Unique inducible filamentous motility identified in pathogenic Bacillus cereus group species
Source: ISME J. 2020 Aug 7;14(12):2997–3010. doi: 10.1038/s41396-020-0728-x (PMC7784679; doi:10.1038/s41396-020-0728-x)
Supplement: Supplementary file 2 — Supplemental Table S1 [file 41396_2020_728_MOESM2_ESM.docx]

**Supplemental Table S1. Bacterial species, primers, and reagents used**

| **Bacteria** | **Description** | **Source** |
| --- | --- | --- |
| *Bacillus mobilis* ML-A2C4 | Isolated strain | This study |
| *Bacillus cereus* ATCC 14579 | Wild type, reference strain | ATCC |
| *Bacillus cytotoxicus* DSM22905 | Wild type, reference strain | DSMZ |
| *Bacillus licheniformis* DSM 13 | Wild type, reference strain | DSMZ |
| *Bacillus megaterium* DSM 32 | Wild type, reference strain | DSMZ |
| *Bacillus mobilis* 0711P9-1 | Wild type, reference strain | KCTC |
| *Bacillus subtilis* DSM 23778 | Wild type, reference strain | DSMZ |
| *Bacillus thuringiensis* DMS2046 | Wild type, reference strain | DSMZ |
| *Bacillus wiedmannii* FSL W8-0169 | Wild type, reference strain | Miller, R.A. et al. 2016 |
| *Bacillus wiehenstephanensis* DSM11821 | Wild type, reference strain | DSMZ |
| *Campylobacter jejuni* 11168 | Wild type, reference strain | Gaynor, E. C. et al. 2004 |
| *Escherichia coli* DH5α | F– Φ80*lacZ*ΔM15 Δ(*lacZYA*-*argF*) U169 *recA1* *endA1 hsdR17* (rK–, mK+) *phoA* *supE44* λ– *thi*-1 *gyrA96* *relA1* | Invitrogen |
| *Bacillus cereus* 407 | Wild type | Houry, A. et al, 2010 |
| *Bacillus cereus* 407 *Δfla* | Flagella locus deletion | Houry, A. et al, 2010 |
| *Bacillus cereus* 407 *ΔmotA* | *motA* deletion | Houry, A. et al, 2010 |
|  |  |  |
| **Primers** | **Sequence** | **Source/Prepared** |
| 16S rRNA Forward | GAGAGTTTGATCCTGGCTCAG | Miao, V.P.W. et al, 1997 |
| 16S rRNA Reverse | CGGCTACCTTGTTACGACTTC | Miao, V.P.W. et al, 1997 |
| *panC* upstream | GAAGAATGCTTATCGTTATACGG | This study |
| *panC* downstream | CATCATTGTGCGAAACATAGATG | This study |
|  |  |  |
| **Plate conditions** | **Description** |  |
| MH | Mueller Hinton (21 g/L) (Oxoid) |  |
| LB | Luria Bertani (25 g/L) (Sigma) |  |
| Tryptone Broth | 10 g/L Tryptone, 5 g/L NaCl |  |
| BHI | Bacto Brain Heart Infusion (35 g/L) (BD) |  |
| MH 0.5X | 10.5 g/L MH |  |
| MH 1.5X | 31.5 g/L MH |  |
| MH 2.0X | 42 g/L MH |  |
| 5% blood agar | Sheep blood (5% v/v) in MH agar |  |
| 15% blood agar | Sheep blood (15% v/v) in MH agar |  |
|  |  |  |
| **Filamentous motility inducing conditions** | **Description** |  |
| Skim milk powder, 10% | 10% w/v |  |
| Unsweetened organic fortified soy beverage | 100% (Natura) |  |
| Skim milk fat free | 100% (Dairyland) |  |
| 1% partly skimmed milk | 100% (Dairyland) |  |
| 2% partly skimmed milk | 100% (Dairyland) |  |
| 3.25% homogenized milk | 100% (Dairyland) |  |
| Creamo half&half | 100% (Dairyland) |  |
| 1% chocolate partly skimmed milk | 100% (Dairyland) |  |
| Sheep blood (defibrinated) | 100% (Dalynn Biologicals) |  |
| *E. coli* DH5α inner membrane | From 0.5 L O/N culture in 1 mL 50% EtOH |  |
| *E. coli* DH5α outer membrane | From 0.5 L O/N culture in 1 mL 50% EtOH |  |
| *C. jejuni* 11168 inner membrane | From 1 L O/N culture in 1 mL 50% EtOH |  |
| *C. jejuni* 11168 outer membrane | From 1 L O/N culture in 1 mL 50% EtOH |  |
| **Filamentous motility inducing conditions (continued)** | **Description** |  |
| L-α-Phosphatidylcholine from egg yolk type XVI-E | 2% w/v in 50% EtOH |  |
| Sterile filtered extracts of human feces (H1C, H2, H3, H4, H5, H6, H9, H10, H11) | 100% |  |
|  |  |  |
| **Filamentous motility non-inducing conditions** | **Description** |  |
| Coconut milk | 100% (Aroy-D) |  |
| Almond milk | 100% (Almond Breeze) |  |
| ATP | 1 mM |  |
| BHI | 10 X |  |
| Bile extract | 0.3% |  |
| Bile salts | 0.1% |  |
| Casamino acids | 30% |  |
| Casein | 2% |  |
| DNA (*C. jejuni* genomic) | 0.9 μg/mL |  |
| Fetal bovine serum | 100% (Gibco) |  |
| Glucose | 40% |  |
| Glycerol | 2 and 20% |  |
| Iron citrate | 1 mM |  |
| Lactose | 2 and 20% |  |
| MEM | 100% (Gibco) |  |
| MH | 10 X |  |
| PEG | 2 and 20% |  |
| Porcine mucin | 0.5 and 5 mg/mL |  |
| SDS | 1 and 5% |  |
| Sodium phosphate | 1 M |  |
| Yeast extract | 25% |  |
