# Supplementary material for: Unique inducible filamentous motility identified in pathogenic Bacillus cereus group species
Source: ISME J. 2020 Aug 7;14(12):2997–3010. doi: 10.1038/s41396-020-0728-x (PMC7784679; doi:10.1038/s41396-020-0728-x)
Supplement: Supplementary file 3 — Supplemental Table S2 [file 41396_2020_728_MOESM3_ESM.docx]

**Supplemental Table S2. *Bacillus* spp. used for phylogenetic analysis and placement of *B. mobilis* ML-A2C4**

| **Species** | **Strain** | **Species Status^a^** | **RefSeq Accession** | **panC Clade ^b^** | **MLST ST ^c^** | **rpoB AT ^d^** |
| --- | --- | --- | --- | --- | --- | --- |
| *B. mobilis* | ML-A2C4 | Published | GCA_003612955.1 | II | 953 | NA |
| *B. albus* | N35-10-2 | Published | GCF_001884185.1 | II | 775 | NA |
| *B. anthracis* | Ames | Published | GCF_000007845.1 | III | 1 | NA |
| *B. cereus s.s.* | ATCC 14579 | Published | GCF_000007825.1 | IV | 921 | 158 |
| *B. cytotoxicus* | NVH 391-98 | Published | GCF_000017425.1 | VII | NA | NA |
| *B. luti* | TD41 | Published | GCF_001884105.1 | II | 764 | NA |
| *B. mobilis* | 0711P9-1 | Published | GCF_001884045.1 | II | 784 | 194 |
| *B. mycoides* | DSM 2048 | Published | GCF_000003925.1 | VI | 116 | 3 |
| *B. nitratireducens* | 4049 | Published | GCF_001884135.1 | VI | 769 | NA |
| *B. pacificus* | EB422 | Published | GCF_001884025.1 | II | 32 | 380 |
| *B. paramycoides* | NH24A2 | Published | GCF_001884235.1 | VI | 780 | NA |
| *B. paranthracis* | Mn5 | Published | GCF_001883995.1 | II | 761 | 125 |
| *B. proteolyticus* | TD42 | Published | GCF_001884065.1 | VI | 765 | NA |
| *B. pseudomycoides* | DSM 12442 | Published | GCF_000161455.1 | I | 83 | 148 |
| *B. thuringiensis* | ATCC 10792 | Published | GCF_002119445.1 | IV | 10 | NA |
| *B. toyonensis* | BCT-7112 | Published | GCF_000496285.1 | V | 111 | 129 |
| *B. tropicus* | N24 | Published | GCF_001884035.1 | II | 771 | NA |
| *B. weihenstephanensis* | WSBC 10204 | Published | GCF_000775975.1 | VI | 196 | 3 |
| *B. wiedmannii* | FSL W8-0169 | Published | GCF_001583695.1 | II | 1081 | 61 |
| *B. bingmayongensis* | FJAT-1383 | Effective | GCF_000299035.1 | NA | 763 | NA |
| *B. gaemokensis* | JCM 15801 | Effective | GCF_000712615.1 | NA | 768 | NA |
| *B. manliponensis* | JCM 15802 | Effective | GCF_000712595.1 | NA | NA | NA |

^a^ The status of the species indicates whether the species have been validated (i.e., Published) or not (i.e., Effective).

^b^ *panC* clade as defined by Guinebretière et al., 2008.

^c^ MultiLocus Sequence Types as defined in the PubMLST *B. cereus* database: <https://pubmlst.org/bcereus/>.

^d^ *rpoB* allelic types as defined by the Milk Quality Improvement Program *rpoB* method and database.
