# Supplementary material for: Unique inducible filamentous motility identified in pathogenic Bacillus cereus group species
Source: ISME J. 2020 Aug 7;14(12):2997–3010. doi: 10.1038/s41396-020-0728-x (PMC7784679; doi:10.1038/s41396-020-0728-x)
Supplement: Supplementary file 4 — Supplemental Table S3 [file 41396_2020_728_MOESM4_ESM.docx]

**Supplemental Table S3. Virulence and antimicrobial resistance gene homologs encoded by *B. mobilis* ML-A2C4**

| **Locus Tag** | **Gene** | **Virulence Gene Product** |
| --- | --- | --- |
| MLA2C4_13355 | *bla-1* | Beta lactamase I |
| MLA2C4_17920 | *bla2* | Beta lactamase II |
| MLA2C4_27295 | *bpsE* | UTP--glucose-1-phosphate uridylyltransferase GalU |
| MLA2C4_27305 / 27315 | *bpsF* | CpsD/CapB family tyrosine-protein kinase |
| MLA2C4_27235 | *bpsH* | LytR family transcriptional regulator |
| MLA2C4_03565 | *cerA / plcB* | Cereolysin A / phospholipase C |
| MLA2C4_03570 | *cerB / sph* | Cereolysin B / sphingomyelinase C |
| MLA2C4_26480 | *clo* | Cereolysin O |
| MLA2C4_27100 | *entA* | Enterotoxin / cell-wall binding protein |
| MLA2C4_09920 | *entFM* | Peptidoglycan endopeptidase |
| MLA2C4_10335 | *fosBx1* | Fosfomycin resistance bacillithiol transferase |
| MLA2C4_16375 | *hblA* | Hemolysin BL binding component precursor |
| MLA2C4_16370 | *hblB* | Hemolysin BL binding component precursor |
| MLA2C4_16385 | *hblC* | Hemolysin BL lytic component L2 |
| MLA2C4_16380 | *hblD* | Hemolysin BL lytic component L1 |
| MLA2C4_18270 | *hlyII* | Hemolysin II |
| MLA2C4_18265 | *hlyR* | Hemolysin II regulatory protein |
| MLA2C4_06830 | *inhA1* | Immune inhibitor A precursor |
| MLA2C4_03540 | *inhA2* | Immune inhibitor A precursor |
| MLA2C4_18260 | *lsaB* | ABC-F subfamily protein |
| MLA2C4_09595 | *nheA* | Non-hemolytic enterotoxin lytic component L2 |
| MLA2C4_09600 | *nheB* | Non-hemolytic enterotoxin lytic component L1 |
| MLA2C4_09605 | *nheC* | Enterotoxin C |
| MLA2C4_19650 | *plcA* | 1-Phosphatidylinositol phosphodiesterase precursor |
| MLA2C4_27700 | *plcR* | Transcriptional activator PlcR |
| MLA2C4_25245 | *vanR-M* | OmpR-family transcriptional activator |
