# Supplementary material for: Unique inducible filamentous motility identified in pathogenic Bacillus cereus group species
Source: ISME J. 2020 Aug 7;14(12):2997–3010. doi: 10.1038/s41396-020-0728-x (PMC7784679; doi:10.1038/s41396-020-0728-x)
Supplement: Supplementary file 9 — Supplemental Methods S1 [file 41396_2020_728_MOESM9_ESM.docx]

**Unique inducible filamentous motility identified in pathogenic *Bacillus* *cereus* group species**

Martha M. Liu ^1^, Shannon Coleman ^1^, Lauren Wilkinson ^1^, Maren L. Smith ^1^, Thomas Hoang ^1^, Naomi Niyah ^2^, Manjari Mukherjee ^2^, Steven Huynh ^3^, Craig T. Parker ^3^, Jasna Kovac ^2^, Robert E.W. Hancock ^1^, Erin C. Gaynor ^1^*

1 Department of Microbiology and Immunology, The University of British Columbia, Vancouver, BC, Canada

2 Department of Food Science, The Pennsylvania State University, University Park, PA, 16802, USA

3 Produce Safety and Microbiology Unit, Western Region Research Center, USDA, Agricultural Research Service, Albany, CA, USA

**SUPPLEMENTAL METHODS S1**

**Whole Genome Sequencing**

Genomic DNA was prepared and sequenced using Pacific Biosciences (PacBio, Menlo Park, CA) RSII with 20-kb SMRTbell libraries as described previously (1) except that *B. mobilis* ML-A2C4 cells were treated with lysozyme overnight at 37 °C. Sequencing of this genomic DNA was also performed on the Illumina MiSeq platform (lllumina, San Diego) using the KAPA LTP library preparation kit (KAPA Biosystems, Wilmington, MA) as previously described (2). A final base call correction and validation of the ML-A2C4 genome was performed upon the PacBio contig and ≥Q20-trimmed MiSeq reads using the reference assembler and the SNP identifier within Geneious software (v11.1) (Biomatters, Ltd., Auckland, NZ).

**Phylogenetic analysis**

For phylogenetic analysis and genotyping, whole genome sequences of ML-A2C4 and representative isolates of 18 putative and 3 effective *B. cereus* group species listed in Supplemental Table S2 were used for single nucleotide polymorphism (SNP) identification using kSNP 3 with an optimal kmer size of 21 (3). Identified core SNPs were used to construct a maximum likelihood tree in RAxML version 8 with 1000 bootstraps, ASC_GTRGAMMA substitution model and Lewis ascertainment bias correction (4). The tree was edited in Figtree version 1.4.3 and Graphic for Mac version 3.1. BTyper (5) version 2.3.1 was used for further genomic characterization of *B. mobilis* ML-A2C4 closed genome, including detection of virulence genes and antimicrobial resistance genes using a method described by Carroll et al., 2017 (5, 6) (Supplemental Table S3), and genotyping based on *panC*, *rpoB* and 7 MLST allele sequences (7-10). Genotyping using *rpoB* sequence typing and MLST identified the *rpoB* allelic type AT0059 with 99.7% identity and 100% coverage as the closest match, and MLST ST 953. Close similarity of ML-A2C4 to the *B. mobilis* type strain was confirmed by both *panC* (97.4% identity) and pairwise average nucleotide identity blast (ANIb) (96.3 pairwise ANIb value) (11) implemented in BTyper version 2.3.1. Antimicrobial and virulence genes were verified as homologs using BLAST.

**Cytotoxicity Testing**

*B. mobilis* ML-A2C4 and type strains of *B. cereus* ATCC 14579 ^T^ , *B. pseudomycoides* DSM 12442^T^, *B. wiedmannii* FSL W8-0169 ^T^, and *B. mobilis* 0711P9-1^T^ were grown in brain heart infusion (BHI) broth at 37°C to stationary phase (12). The stationary phase was determined for each individual isolate as previously reported (13). Culture supernatants were collected in two independent replicates at the end of the incubation period using centrifugation (5 min at 16000 g), and were stored at −80°C until intoxication. HeLa and CaCo-2 cells were seeded at a density of 8 x10^4^ cells/cm^2^ in a 96-well microtiter plate and grown with Eagle’s minimum essential medium supplemented with 10% (HeLa cells) or 20% (CaCo-2 cells) fetal bovine serum (EMEM+FBS) for 24 h. Cytotoxicity experiments were conducted by replacing the cell medium with fresh EMEM+FBS, supplemented with 5% v/v (for HeLa cells) or 15% v/v (for CaCo-2 cells) of bacterial supernatants collected in the first or second independent experimental replicate, respectively. EMEM+FBS was used as a blank, EMEM+FBS containing just 5% BHI was used as a negative control, and EMEM containing 0.05% Triton X-100 was used as a positive control for cytotoxicity. *B. cereus* ATCC 14579^T^ and *B. pseudomycoides* DSM 12442^T^ were used as additional positive controls for isolate cytotoxicity, as they have been characterized as highly cytotoxic in a previous study (7). After 15-min exposure of both HeLa and CaCo-2 cells to bacterial supernatant at 37°C and 5% CO_2_, 10 μL of WST-1 dye solution (Roche) was added to each test well, followed by 25-min incubation (for HeLa cells) or 90-min incubation (for CaCo-2 cells) under the same conditions. After the completed incubation with the WST-1 dye, the plates were subjected to orbital shaking for 30 s, the absorbance of the suspension in each well of a microtiter plate was read at 450 nm and 690 nm. The latter was used to correct for the background signal by means of subtraction from the former. The viability of cells exposed to bacterial supernatants was calculated by taking a ratio of corrected absorbance to that of the negative control (EMEM+BHI) and converting an obtained value to percentage. A total of six to twelve replicates were conducted per cell line.

**Substrate set-up for plating**

*C. jejuni* lawn plates were prepared using a two day spread plating technique. On day one, 200 µL of a 0.005 OD_600_ diluted overnight culture was spread onto each agar plate and incubated microaerobically overnight in a Sanyo tri-gas incubator at 38°C. On day two, the cells in each plate were spread again without additional input and incubated overnight under the same conditions. Plates were stored aerobically at room temperature for one to five days until needed. *C. jejuni* is microaerophilic and thermophilic, therefore cells on these stored plates were expected to be metabolically inactive and/or dead. Live *C. jejuni* lawn plates were used immediately after the second incubation without storage at room temperature. Small *C. jejuni* spots on plates were made by inoculating 10 μL droplets of overnight culture diluted to 0.005 OD_600_, then incubating overnight. All types of milk (Supplemental Table S1) were sterilized by autoclaving prior to spreading onto plates. Whole blood was also split into cells and plasma by centrifuging twice at 1000 rpm (179 rcf) for 10 min in a tabletop centrifuge using a swing bucket rotor. The clear plasma was aliquoted and used as is, while the cell fraction was re-diluted with MEM to the starting volume. Plates containing milks, blood, and plasma were prepared by swirling 1.5 mL of the substrate on the surface of an agar plate until fully and uniformly coated then allowing the liquid to dry onto the agar surface. 5% and 15% blood agar plates were prepared by adding blood to the correct final concentration in warm agar before pouring plates. Lawns of heat-killed cells were prepared by pipetting 0.4 mL of autoclaved cell suspensions that were concentrated to 1, 5 or 15 OD_600_ from overnight bacterial broth cultures onto the agar plate. Phosphatidylcholine (PC) plates prepared by spread plating 0.5 mL of 2% PC in 50% EtOH (prepared from 4% w/v PC in 100% EtOH), and control plates were spread with 0.5mL of 50% EtOH. Nine human fecal extracts (H1C, H2, H3, H4, H5, H6, H9, H10, and H11) were obtained from Liu et.al. 2018 (14) as detailed in the approved ethics application H18-02870. These extracts were prepared by filter sterilizing fecal material diluted 1:1 to 1:1.5 w:v in water. 100 μL of extract was pipetted onto agar and the droplet was spread out to ~1.5 - 1.8 cm diameter circles . Crude bacterial membranes were isolated using a modified version of a sucrose gradient protocol (refer to: <http://cmdr.ubc.ca/bobh/method/outer-membrane-preparation-one-step-sucrose-gradient-procedure/>) and detailed in Supplemental Text SXX. Bacterial membrane lawns were made by pipetting 100 μL of diluted membrane (in 25% EtOH) onto agar plates, and control plates used 100 μL of 25% EtOH only. All plates with spread or spot plated materials were prepared one to five days before start of experiments and stored at room temperature.

**Bacterial Outer Membrane Preparation**

Bacterial membrane preparations were isolated using a modified version of the sucrose gradient protocol described in http://cmdr.ubc.ca/bobh/method/outer-membrane-preparation-one-step-sucrose-gradient-procedure/. Briefly, *C. jejuni* and *E. coli* cells from 1 L of overnight shaken culture were resuspended in 20% sucrose in 10 mM Tris pH 8.0, treated with 100 units/mL DNase (Invitrogen) for 20 min at room temperature and stored frozen. Bacterial suspensions were thawed, French pressed twice, and pelleted at 3K rpm (2K rcf) for 10 min at 4°C. Supernatants were layered on top of 60% and 70% sucrose gradients and ultracentrifuged at 23K rpm (95K rcf) for 18 h at 4°C. The inner (between 20% and 60% sucrose) and outer (between 60% and 70% sucrose) membrane layers were collected, diluted 1:2 with dH_2_O, and pelleted at 47K rpm (227K rcf) for 1 h at 4°C. Membranes were resuspended in dH_2_O before addition of an equal volume of EtOH and stored frozen. Prior to use membrane suspensions were diluted 1:1 with dH_2_O.

**RNA sequencing**

For each sample 1.8 OD _600_ of cells collected from two to four plates were scraped into a tube containing RNA Protect Bacteria Reagent (QIAGEN), pelleted, and stored at -80°C. Pellets were resuspended in 1 ml buffer RLT (QIAGEN) supplemented with 1/100 β-mercaptoethanol and transferred to tubes containing lysing matrix B (MP Biomedicals). Samples were bead bashed in the Bead Ruptor 24 (Omni International) once at speed 7 for 30 s and twice at speed 8 for 30 s, with chilling on ice in between. After a brief spin, the supernatant was mixed with 700 μL 70% EtOH. RNA isolation then proceeded according to the manufacturer’s instructions using the RNeasy Mini Kit (QIAGEN). Eluted RNA was further purified with the TURBO DNA-free kit (Thermo Fisher). RNA samples were depleted of rRNA using the RiboZero Bacteria Kit (Illumina). Libraries of cDNA were prepared using the KAPA Stranded Total RNA Kit (Kapa Biosystems) and sequenced on an Illumina HiSeq 2500 by the UBC Sequencing and Bioinformatics Consortium. A total of 4.6 to 13.2 million sequences were obtained per replicate, and 76.4% to 93.4% of reads in each replicate were mapped to the ML-A2C4 genome.

**RNA-Seq Data Analysis**

RNA-Seq fastq reads were determined using FastQC v0.11.5 and MulitQC v1.6, and RNA reads for all samples were mapped to the *B. mobilis* MLA2C4 genome with STAR version 2.6.0c. Read counts for individual genes were obtained using HTSeq-count v0.9.1. Significantly differentially expressed genes (false discovery rate ≤0.05 and fold change ≥±2) were identified using DESEQ2 1.20.0. Because resources for functional enrichment analysis were not available for this relatively newly described *B. mobilis* strain, ML-A2C4 genes, based on protein product, were converted to *B. cereus* orthologs using the open source resource, OrthoFinder (<https://doi.org/10.1186/s13059-015-0721-2>). Differentially expressed (DE) genes between filamentous growth on 10% skim milk and PC lawns and control conditions (as orthologs) were used as inputs for enrichment analysis using the Gene Ontology biological process (<http://pantherdb.org/>) and KEGG. Comparison of DE genes between filamentous growth on PC vs. control and 10% milk vs. control (including PCA plot, heatmap, and colors representing fold change) were prepared using R. The proportional Venn Diagram was made using the online software Meta-Chart Venn Diagram maker (<https://www.meta-chart.com/venn>). *B. cereus* orthologs of *B. mobilis* DE genes were compared to *B. cereus* swarming vs. nonswarming DE genes from Salvetti *et.al*. (2011) using hypergeometric overlap. Overlap comparisons were made using the userListEnrichment() function from WGCNA (https://www.ncbi.nlm.nih.gov/pubmed/16646834).

**References**

1. Miller WG, Yee E, Chapman MH, Smith TP, Bono JL, Huynh S, et al. Comparative genomics of the Campylobacter lari group. Genome Biol Evol. 2014;6(12):3252-66.

2. Parker CT, Cooper KK, Huynh S, Smith TP, Bono JL, Cooley M. Genome Sequences of Eight Shiga Toxin-Producing Escherichia coli Strains Isolated from a Produce-Growing Region in California. Microbiol Resour Announc. 2018;7(1).

3. Gardner SN, Slezak T, Hall BG. kSNP3.0: SNP detection and phylogenetic analysis of genomes without genome alignment or reference genome. Bioinformatics. 2015;31(17):2877-8.

4. Stamatakis A. RAxML version 8: a tool for phylogenetic analysis and post-analysis of large phylogenies. Bioinformatics. 2014;30(9):1312-3.

5. Carroll LM, Kovac J, Miller RA, Wiedmann M. Rapid, High-Throughput Identification of Anthrax-Causing and Emetic Bacillus cereus Group Genome Assemblies via BTyper, a Computational Tool for Virulence-Based Classification of Bacillus cereus Group Isolates by Using Nucleotide Sequencing Data. Appl Environ Microbiol. 2017;83(17).

6. Carroll LM, Wiedmann M, den Bakker H, Siler J, Warchocki S, Kent D, et al. Whole-Genome Sequencing of Drug-Resistant Salmonella enterica Isolates from Dairy Cattle and Humans in New York and Washington States Reveals Source and Geographic Associations. Appl Environ Microbiol. 2017;83(12).

7. Miller RA, Jian J, Beno SM, Wiedmann M, Kovac J. Intraclade Variability in Toxin Production and Cytotoxicity of Bacillus cereus Group Type Strains and Dairy-Associated Isolates. Appl Environ Microbiol. 2018;84(6).

8. Guinebretiere MH, Thompson FL, Sorokin A, Normand P, Dawyndt P, Ehling-Schulz M, et al. Ecological diversification in the Bacillus cereus Group. Environ Microbiol. 2008;10(4):851-65.

9. Jolley KA, Maiden MC. BIGSdb: Scalable analysis of bacterial genome variation at the population level. BMC Bioinformatics. 2010;11:595.

10. Ivy RA, Ranieri ML, Martin NH, den Bakker HC, Xavier BM, Wiedmann M, et al. Identification and characterization of psychrotolerant sporeformers associated with fluid milk production and processing. Appl Environ Microbiol. 2012;78(6):1853-64.

11. Richter M, Rossello-Mora R. Shifting the genomic gold standard for the prokaryotic species definition. Proc Natl Acad Sci U S A. 2009;106(45):19126-31.

12. Carroll LM, Wiedmann M, Mukherjee M, Nicholas DC, Mingle LA, Dumas NB, et al. Characterization of Emetic and Diarrheal Bacillus cereus Strains From a 2016 Foodborne Outbreak Using Whole-Genome Sequencing: Addressing the Microbiological, Epidemiological, and Bioinformatic Challenges. Front Microbiol. 2019;10:144.

13. Fermanian C, Lapeyre C, Fremy JM, Claisse M. Production of diarrheal toxin by selected strains of Bacillus cereus. Int J Food Microbiol. 1996;30(3):345-58.

14. Liu MM, Boinett CJ, Chan ACK, Parkhill J, Murphy MEP, Gaynor EC. Investigating the Campylobacter jejuni Transcriptional Response to Host Intestinal Extracts Reveals the Involvement of a Widely Conserved Iron Uptake System. MBio. 2018;9(4).
